# Pulmonary *Streptomyces* Infection in Patient with Sarcoidosis, France, 2012

**DOI:** 10.3201/eid1811.120797

**Published:** 2012-11

**Authors:** Etienne Riviere, Didier Neau, Xavier Roux, Nicolas Lippa, Julien Roger-Schmeltz, Patrick Mercie, Maïté Longy-Boursier

**Affiliations:** University Hospital Center of Bordeaux, Bordeaux, France; and Bordeaux Segalen University, Bordeaux

**Keywords:** Streptomyces, Actinomycetales, pneumonia, sarcoidosis, immune deficiency, antibiotics, antimicrobial drugs, mycetoma, actinomycetoma, saprophyte, splenectomy, bacteria, France

**To the Editor**: *Streptomyces* spp. are aerobic, gram-positive bacteria of the order Actinomycetales, known for their ability to produce antimicrobial molecules such as streptomycin. *Streptomyces* spp.*,* usually saprophytic to humans, can cause local cutaneous fistulized nodules known as actinomycetoma or mycetoma. Severe invasive infections have seldom been reported, but most cases reported have occurred in immunocompromised patients (*1*–*5*). We report a case of invasive pulmonary infection caused by a *Streptomyces* sp. in a splenectomized patient with sarcoidosis.

In 2003, multiorgan sarcoidosis was diagnosed in a man, 57 years of age; the disease involved lungs, skin, joints, and lymph nodes. Corticosteroids were initially given but quickly discontinued because of a severe psychiatric reaction. In 2007, a splenectomy was performed on this patient to remove an intestinal obstruction caused by a severely enlarged spleen, identified as a specific localization of sarcoidosis.

In April 2008, the patient was admitted to the internal medicine unit of Saint-André Hospital in Bordeaux, France with fever (38.9°C/102°F), progressive asthenia, anorexia, weight loss, productive cough, and New York Heart Association grade III dyspnea. Bilateral basal crackles could be heard in the lungs; physical examination findings were otherwise within normal limits. Biological tests showed inflammatory syndrome with elevated C-reactive protein (74 mg/L, reference value <5 mg/L) without any other consequential abnormality. Gamma globulin levels were normal. A chest radiograph showed bilateral interstitial infiltrate. A computed tomography scan of the chest confirmed an interstitial micronodular infiltrate with thickening of the peribronchovascular interstitium, associated with paratracheal and left anterior mediastinal supracentimetric lymph nodes.

To determine whether this infiltrate was linked to sarcoidosis, tuberculosis, or another opportunistic infection, bronchoscopy and bronchoalveolar lavage (BAL) were performed and showed multiple submucous nodules of the left superior bronchus. Biopsy samples contained epithelioid granulomas and a nonspecific, amorphous eosinophilic material without focal necrosis, but no bacteria, by using periodic acid–Schiff, Ziehl–Neelsen, and auramine-rhodamine stains. BAL culture isolated a *Streptomyces* sp. (2 × 10^5^ CFU/mL) but no other pathogens.

Treatment with intravenous imipenem (2 g/day for 14 days) and amikacin (1 g/day for 3 days) was initiated. After antimicrobial susceptibility tests, the treatment was changed to oral rifampin (1.2 g/day) and ciprofloxacin (1.5 g/day) for 6 months. After 3 days of treatment, clinical signs and symptoms resolved; a thoracic computed tomography scan performed 6 months later showed complete regression of pulmonary infiltrates. Bronchoscopy at that time showed no nodules, and BAL culture showed no pathogens.

*Streptomyces* spp*.* are widespread environmental bacteria that rarely cause severe invasive infections. During our literature search, we found 21 cases of invasive *Streptomyces* infections, including 8 pulmonary infections. A contributing factor was found for all cases: immunosuppression linked to HIV infection (*1*), antineoplastic chemotherapy (*2*), Crohn disease (*3*), use of oral (*4*) or inhaled corticosteroids (*5*), and presence of foreign material such as a central venous catheter (*6*) or a prosthetic aortic valve (*7*).

Specific features of pulmonary *Streptomyces* infection are summarized in the Technical Appendix Table. Death related to such an infection is mostly dependent on the underlying disease associated with *Streptomyces* infection. Deaths have not been linked to *Streptomyces* infections described in the literature when *Streptomyces* sensibility testing was performed and treatment length recommendations were followed.

To understand how the patient was infected with a *Streptomyces* sp., we explored 2 possibilities. First, sarcoidosis induces immune deficiency (*8*). This phenomenon is clinically well known as anergy to tuberculin or other immunogenic haptens after subcutaneous injections. Expansion of regulatory T lymphocytes (*8*) and attenuated myeloid dendritic cell functions (*9*) decrease cellular immunity efficiency and increase infectious episodes in affected patients. Second, splenectomy can increase susceptibility to infection, such as bloodstream infections with encapsulated bacteria or opportunistic infections with *Campylobacter jejuni*, *Pneumocystis jiroveci*, or *Babesia* spp. The lung infection with *Streptomyces* in the patient described was not acquired through the bloodstream, but through direct airway contact. However, we could not exclude other immune mechanisms not related to blood, such as dysregulation or lack of some lymphocyte populations.

A pathologic feature of pulmonary infections with *Streptomyces* spp. is the presence of granulomas sometimes associated with focal necrosis. This feature makes differentiating infection with these species from that of tuberculosis difficult. Bacterial culture is often used to confirm the diagnosis. Histologic differences between the 2 entities are not well defined because of the rarity of invasive *Streptomyces* infections. In our observation of this patient, histologic examination revealed granulomas potentially linked to sarcoidosis and a nonspecific, amorphous eosinophilic material that was not caseous necrosis. Both lesions could have also resulted from the *Streptomyces* infection. For further identification, Dunne et al. added the presence of sulfur granules to the specific histological description of *Streptomyces* infection (*1*).

An overall literature review for results of in vitro testing for *Streptomyces* spp. identified a common susceptibility to aminoglycosides, macrolides, imipenem, or trimethoprim/sulfamethoxazole. This finding suggests that the first-line treatment against invasive *Streptomyces* infections should begin with imipenem and aminoglycosides for at least 6 weeks (Technical Appendix Table). Quinolones have an immunomodulatory effect that might be therapeutic in patients with disease-induced immunosuppression such as sarcoidosis or after splenectomy (*10*). In conclusion, invasive *Streptomyces* infection of the lungs should be included in differential diagnoses of interstitial pneumonia in immunocompromised patients.

Technical Appendix Contributing factors, presence or absence of fever, results of CT scans of the chest, diagnostic methods and results, and treatment of patients with pulmonary *Streptomyces* spp. Infection.
