# Supplementary material for: Pulmonary Streptomyces Infection in Patient with Sarcoidosis, France, 2012
Source: Emerg Infect Dis. 2012 Nov;18(11):1907–9. doi: 10.3201/eid1811.120797 (PMC3559136; doi:10.3201/eid1811.120797)
Supplement: Technical Appendix — Contributing factors, presence or absence of fever, results of CT scans of the chest, diagnostic methods and results, and treatment of patients with pulmonary Streptomyces spp. Infection. [file 12-0797-Techapp-s1.pdf]

Article DOI: http://dx.doi.org/10.3201/eid1811.120797

Pulmonary *Streptomyces* Infection in Patient with Sarcoidosis, France, 2012

Technical Appendix

Table. Pulmonary *Streptomyces* infection in a patient with sarcoidosis and case-patients studied in review of literature, France, 2012

Age, y/sex	Contributing factor	Fever	Results of chest CT scan	Diagnosis	*Streptomyces* sp.	Antibacterial treatment	Treatment duration, wks	Reference
57/M	Sarcoidosis, splenectomy	Yes	Micronodular interstitial infiltrate with mediastinal supracentimetric lymph nodes	BAL/culture	Unknown	Imipenem and amikacin, then rifampin and ciprofloxacin	24	This case
43/M	HIV infection for 8 y	Yes	Multiple lung nodules	Lung biopsy specimen	Unknown	Ceftriaxone, then TMP/SMZ, then clarithromycin	>24	Dunne et al. (*1*)
21/F	AML/chemotherapy	No	Multiple lung nodules	Fine-needle aspiration/culture	*maritimus or olivaceus*	Minocyclin and clarithromycin and moxifloxacin	2 (died)	Kapadia et al. Case 1 (*4*)
23/F	SLE/corticosteroids	No	Lung nodule and mediastinal lymph nodes	Lung biopsy specimen	*albus*	None (surgery: excised nodule)	NA	Kapadia et al. Case 2 (*4*)
18/M	Burkitt lymphoma/ chemotherapy	No	Multiple lung nodules	Lung biopsy specimen	Unknown	None (surgery: excised nodule)	NA	Kapadia et al. Case 6 (*4*)
52/F	Inhaled corticosteroids	Yes	Multiple alveolar-type limited fibrotic lesions and bronchiectasies	BAL/culture	*lanatus*	Ceftriaxone, then TMP/SMZ, then clarithromycin	24	Kofteridis et al. (*5*)
35/M	HIV infection	Yes	Alveolar-type infiltration	Sputum sample, BAL/culture	Unknown	Piperacilline and tazobactam, then imipenem	Unknown	Ahmed et al. (*11*)
30/M	HIV infection	Yes	Multiple lung nodules in an interstitial infiltrate	BAL/culture	Unknown	Cefuroxim and amikacin, then amoxicillin and clavulanate	6	Caron et al. (*12*)
50/M	None	Yes	Alveolar-type infiltration	Blood cultures	Unknown	Penicillin, sulfasalazine, streptomycin, aureomycin, and terramycin	6	Kohn et al. (*13*)

*CT, computed tomography; TMP/SMZ, trimethoprim/sulfamethoxazole; AML, acute myeloid leukemia; SLE, systemic lupus erythematosus; NA, not applicable; BAL, bronchoalveolar lavage.

References

1. Dunne EF, Burman W, Wilson M. *Streptomyces* pneumonia in a patient with human immunodeficiency virus infection: case report and review of the literature on invasive *Streptomyces* infections. Clin Infect Dis. 1998;27:93–6. PubMed http://dx.doi.org/10.1086/514612

2. Moss WJ, Sager JA, Dick JD, Ruff A. *Streptomyces bikiniensis* bacteremia. Emerg Infect Dis. 2003;9:273–4. PubMed http://dx.doi.org/10.3201/eid0902.020275

3. Ekkelenkamp MB, de Jong W, Hustinx W, Thijsen S. *Streptomyces thermovulgaris* bacteremia in Crohn's disease patient. Emerg Infect Dis. 2004;10:1883–5. PubMed http://dx.doi.org/10.3201/eid1010.040300

4. Kapadia M, Rolston KVI, Xiang Han XY. Invasive *Streptomyces* infections: six cases and literature review. Am J Clin Pathol. 2007;127:619–24. PubMed http://dx.doi.org/10.1309/QJEBXP0BCGR54L15

5. Kofteridis DP, Maraki S, Scoulica E, Tsioutis C, Maltezakis G, Gikas A. *Streptomyces* pneumonia in an immunocompetent patient: a case report and literature review. Diagn Microbiol Infect Dis. 2007;59:459–62. PubMed http://dx.doi.org/10.1016/j.diagmicrobio.2007.06.009

6. Carey J, Motyl M, Perlman DC. Catheter-related bacteremia due to *Streptomyces* in a patient receiving holistic infusions. Emerg Infect Dis. 2001;7:1043–5. PubMed http://dx.doi.org/10.3201/eid0706.010624

7. Mossad SB, Tomford JW, Stewart R, Ratliff NB, Hall GS. Case report of *Streptomyces* endocarditis of a prosthetic aortic valve. J Clin Microbiol. 1995;33:3335–7. PubMed

8. Iannuzzi MC, Rybicki BA, Teirstein AS. Sarcoidosis. N Engl J Med. 2007;357:2153–65. PubMed http://dx.doi.org/10.1056/NEJMra071714

9. Mathew S, Bauer KL, Fischoeder A, Bhardwaj N, Oliver SJ. The anergic state in sarcoidosis is associated with diminished dendritic cell function. J Immunol. 2008;181:746–55. PubMed

10. Dalhoff A, Shalit I. Immunomodulatory effects of quinolones. Lancet Infect Dis. 2003;3:359–71. PubMed http://dx.doi.org/10.1016/S1473-3099(03)00658-3

11. Ahmed AJ, Ali ST, Weinbaum D, Goldberg E. *Streptomyces* infection in AIDS presenting with pneumonia and monarthritis. Inf Dis Clin Pract. 1996;5:207–8.

12. Caron F, Borsa-Lebas F, Boiron P, et al. *Streptomyces* sp as a cause of nodular pneumonia in a HIV infected patient? Med Microbiol Lett. 1992;1:297–303.

13. Kohn PM, Tager M, Siegel ML, Ashe, R. Aerobic *Actinomyces* septicemia: report of a case. N Engl J Med. 1951;245:640–4. PubMed http://dx.doi.org/10.1056/NEJM195110252451703
